# Feasibility of a fast-track randomized controlled trial of cell-free and concentrated ascites reinfusion therapy for patients with refractory malignant ascites

**DOI:** 10.1186/s12885-022-09336-3

**Published:** 2022-02-28

**Authors:** Naosuke Yokomichi, Kengo Imai, Masaki Sakamoto, Masashi Horiki, Toshihiro Yamauchi, Satoru Miwa, Satoshi Inoue, Yu Uneno, Hidekazu Suzuki, Toru Wada, Yuri Ichikawa, Tatsuya Morita

**Affiliations:** 1 Division of Palliative and Supportive Care, Seirei Mikatahara General Hospital, 3453 Mikatahara-cho, Kita-ku, Hamamatsu, Shizuoka 433-8558 Japan; 2 Seirei Hospice, Seirei Mikatahara General Hospital, Hamamatsu, Japan; 3 Department of Surgery, Nagoya Tokushukai General Hospital, Kasugai, Japan; 4 Departments of Gastroenterology and Hepatology, Itami City Hospital, Itami, Japan; 5 Department of Therapeutic Oncology, Graduate School of Medicine, Kyoto University, Kyoto, Japan; 6 Division of Medical Engineering, Seirei Mikatahara General Hospital, Hamamatsu, Japan

**Keywords:** Cell-free and concentrated ascites reinfusion therapy, Malignant ascites, Paracentesis

## Abstract

**Background:**

Malignant ascites often causes discomfort in advanced cancer patients. Paracentesis is the most common treatment modality, but it requires frequently repeated treatment. Cell-free and concentrated ascites reinfusion therapy (CART) may prolong the paracentesis interval, but controlled trials are lacking. We assessed the feasibility of a randomized controlled trial of CART vs. paracentesis alone for patients with refractory malignant ascites.

**Methods:**

This study was an open-label, fast-track, randomized controlled, feasibility trial. Patients admitted to four designated cancer hospitals who received no further anticancer treatments were eligible. Patients were randomly assigned 1:1 to a CART arm or control (simple paracentesis) arm. The feasibility endpoint was the percentage of patients who completed the study intervention. Secondary endpoints included paracentesis-free survival, patient’s request on the questionnaire for paracentesis (PRO-paracentesis)-free survival (the period until the patients first reported that they would want paracentesis if indicated), and adverse events.

**Results:**

We screened 953 patients for eligibility. Of 61 patients with refractory malignant ascites, 21 patients were determined as eligible. Finally, 20 patients consented and were allocated; 18 patients (90%, 95% CI: 68.3–98.8) completed the study intervention. All patients had an ECOG performance status of 3 or 4. The median drained ascites volume was 3,200 mL in the CART arm and 2,500 mL in the control arm. In the CART arm, the median reinfused albumin volume was 12.6 g. Median paracentesis-free survivals were 5 days (95% CI: 2–6) in the CART arm, and 6 days (3–9) in the control arm. Median PRO-paracentesis-free survivals were 4 days (2–5) and 5 days (1–9), respectively. A total of 73% of patients received paracentesis within 2 days from their first request for the next paracentesis. One patient in the CART arm developed Grade 1 fever.

**Conclusions:**

A fast-track randomized controlled trial of CART for patients with malignant ascites is feasible. The efficacy and safety of CART should be assessed in future trials. PRO-paracentesis-free survival may be a complementary outcome measure with paracentesis-free survival in future trials.

**Trial registration:**

Registered at University Hospital Medical Information Network Clinical Trial Registry as UMIN000031029. Registered on 28/01/2018.

**Supplementary Information:**

The online version contains supplementary material available at 10.1186/s12885-022-09336-3.

## Introduction

Malignant ascites is defined as the abnormal accumulation of fluid in the peritoneal cavity as a consequence of cancer [1–5]. The development of malignant ascites is most frequent in ovarian cancer (38%), followed by pancreaticobiliary, and other gastrointestinal cancers [6]. The median survival of patients with malignant ascites is less than 6 months [6]. Patients with malignant ascites often suffer from distressing symptoms, such as abdominal distension, dyspnea, anorexia, nausea and vomiting, and abdominal pain, especially in their end-of-life stage [1–8]. Control of such symptoms is often difficult when the disease becomes refractory to standard antitumor or molecular-targeted therapies [9–13].

Although there are various treatment modalities for malignant ascites, including paracentesis, pharmacological therapy such as diuretics, peritoneovenous shunts, the intraperitoneal administration of cytotoxic or targeted agents, and intraperitoneal corticosteroids, evidence supporting the efficacy and safety of each approach is weak [1–5, 14, 15]. Paracentesis is still the most common treatment modality used for alleviating the symptom burden from malignant ascites [9, 16]. While paracentesis provides rapid and temporary symptom relief for 90% of patients [2], it requires frequently repeated treatment, e.g., every 10 days, to maintain symptom control because of the reaccumulation of ascites [14, 15]. The optimal procedure is often a balance between the potential for symptom improvement and the known risks of adverse events such as hypotension, renal impairment, perforation, and loss of protein [1–5].

The reinfusion of concentrated ascitic fluid was first proposed in the 1970s for patients with cirrhotic ascites [17–19]. Several randomized controlled trials demonstrated that, by reinfusing autologous proteins in ascites, this treatment was as effective as total paracentesis with albumin infusion for patients with cirrhotic ascites, while it led to a higher risk of fever [20–23]. Cell-free and concentrated ascites reinfusion therapy (CART) was one of the modified procedures for the reinfusion of concentrated ascitic fluid, proposed in 1977 [24–32]. This was the first procedure adopted for malignant ascites with selective filtration to remove malignant cells. Since 1981, CART has been an approved treatment for refractory ascites with malignancy covered by national health insurance in Japan. Despite the widespread use of CART for malignant ascites throughout the country, controlled trials to determine the efficacy and safety of CART for malignant ascites are lacking. Our previous retrospective cohort study revealed that the time to the next paracentesis in patients with malignant ascites who received CART was 28 days, which is twice as long as that of patients treated with paracentesis alone in existing studies [14, 15, 25]. Mild and transient fever occurred in 5 to 12% of patients treated with CART, and severe adverse events were rare [25–28]. A randomized clinical trial is warranted to confirm the efficacy and safety of CART for malignant ascites.

Also, endpoints to determine the efficacy have not been fully established. Although the paracentesis interval is one of the common outcome measures, it could be affected or manipulated, especially in a non-blinded trial [13–15]. It might be better to use it in combination with patient-reported measures, such as the symptom intensity and request for paracentesis, or an objective surrogate for ascites volume, such as the abdominal circumference.

The primary aim of this study was to assess the feasibility of a randomized controlled trial of CART vs. paracentesis alone for malignant ascites. Additionally, we explored potential outcome measures for ascites treatment.

## Methods

This study was an open-label, fast-track, randomized controlled, feasibility trial comparing the effect of CART vs. paracentesis alone for patients with refractory malignant ascites [33, 34]. It was conducted from February 19th, 2018 to July 13th, 2020.

The study was performed in accordance with the ethical standards of the Helsinki Declaration and the ethical guidelines for epidemiologic research of the Ministry of Health, Labour and Welfare of Japan. The protocol, procedures, information sheets, consent forms, and questionnaires were approved by the Institutional Review Board of each site. Patients gave written informed consent before enrollment. It was registered at University Hospital Medical Information Network Clinical Trial Registry (UMIN-CTR) as UMIN000031029. CART costs were reimbursed by national health insurance.

### Participants

This study recruited participants from two palliative care units and two hospital palliative care teams of four designated cancer hospitals. Inclusion criteria were: aged 20 years or older with a diagnosis of metastatic or locally advanced cancer with malignant ascites, received paracentesis within 3 weeks and considered that repeated paracentesis would necessary for symptom control, admitted to participating institutes, and received no further anticancer treatments. Exclusion criteria were: life expectancy of two weeks or less, received chemotherapy, immunotherapy, or abdominal radiotherapy within 28 days, systemic or intra-abdominal infection, fever higher than 38 degrees Celsius, systolic blood pressure <  = 80 mmHg, serum hemoglobin level < 6 mg/dL, serum creatinine level >  = 2.0 ug/dL, serum total bilirubin >  = 5.0 mg/dL, past history of hepatic encephalopathy, cognitive impairment, and no peripheral venous route.

### Intervention

After enrollment, patients received allocated treatment: CART or simple paracentesis, when clinically indicated, i.e., driven mainly by worsening of the patient’s symptoms due to ascites accumulation. In both arms, ascites was removed under local anesthesia and mobilized by gravity. Drainage volumes of patients in each arm were decided by the primary responsible palliative care physician according to the national clinical guideline in which experts proposed 1 to 3 L of paracentesis as a balanced procedure [3].

Following the removal of ascitic fluid, patients in the CART arm received reinfusion of removed ascitic fluid. After ascites drainage, removed ascitic fluid was filtered and concentrated using AHF-MOW and AHF-UP (Asahi Kasei Medical, Tokyo, Japan), which remove bacteria and malignant cells and concentrate materials with a molecular weight of more than 30,000 including autologous albumin. The filtered and concentrated ascetic fluid was reinfused into the patient via a peripheral vein at 50 – 100 mL per hour. We injected 400 mg of acetaminophen intravenously before reinfusion for the prophylaxis of fever.

Patients in the control (waiting list) arm received CART after the study when the next paracentesis was needed.

### Endpoints

Our primary objective was to determine the feasibility of conducting this randomized trial to compare CART vs. paracentesis. The primary feasibility endpoint was the percentage of the patients who completed the study intervention among those patients who consented.

The secondary endpoints included paracentesis-free survival, patient’s request on the questionnaire for paracentesis (PRO-paracentesis)-free survival, and adverse events. Paracentesis-free survival was defined as the time from the day of allocated treatment (day 1) to the day of the next paracentesis or death, whichever occurred first. PRO-paracentesis-free survival was a patient-reported outcome measure defined as the time from day 1 to the day when the patient first reported on the questionnaire that they would want paracentesis if indicated, received the next paracentesis, or died, whichever occurred first.

We further explored multiple potential outcome measures of ascites treatments: the changes of patient reported physical symptom intensities, i.e., the total score of the Edmonton Symptom Assessment System: Ascites Modification (ESAS:AM) and a separate single question of abdominal distension numerical rating scale (NRS), and the change of abdominal circumference.

### Outcome measures

#### Patient’s request for paracentesis on the questionnaire

Patients answered yes or no to the question “If indicated, would you want paracentesis now?” on the questionnaire.

#### Abdominal distension numerical rating scale (NRS)

Patients rated the current intensity of abdominal distension using an 11-point numerical rating scale, ranging from 0 (none) to 10 (worst possible).

#### Edmonton Symptom Assessment System: Ascites Modification (ESAS:AM)

ESAS:AM includes 11 items that consist of nine items of the original ESAS and two additional ascites-related items (‘‘abdominal distension’’ and ‘‘mobility’’) [7, 35]. Patients rated the intensity of each symptom at a point in time (current) using an 11-point numerical rating scale, ranging from 0 (symptom absent or best) to 10 (worst possible). The validity and reliability of the Japanese version of ESAS:AM were previously confirmed [36].

#### Abdominal circumference

Abdominal circumference was measured by the attending nurse of the day at the level of the umbilicus with the patient in a supine position [37].

### Procedures

We followed patients from the day of the allocated treatment (day 1) to the day of the next paracentesis or day 14, whichever occurred first. Outcome measurement and analyses were done for the allocated treatment only.

At enrollment, we obtained data about patient demographics such as age, sex, ECOG performance status, primary tumor site, presence of metastasis in the liver and lung, co-treatments (opioid, diuretics, and artificial hydration), serum laboratory data investigated within a week (total protein, albumin, total bilirubin, and creatinine), and ascites laboratory data investigated within 8 weeks (red blood cell count and albumin). Ascites was defined as hemorrhagic if the ascitic red blood cell count was more than 10,000/μL [38].

During the 14-day follow-up period, patients filled in the questionnaire on their request for paracentesis and abdominal distension NRS once a day, and ESAS:AM on days 1, 2, 4, 6, and 8 at 10 to 12 o’clock. Nurse or family caregiver assistance was allowed in the case that patients found it difficult to fill in the questionnaire by themselves (proxy rating was not allowed). Abdominal circumference was measured once a day at 10 to 12 o’clock.

On day 14, the primary responsible palliative care physician recorded the day of the next paracentesis or death during the observation period. Known adverse events, i.e., fever, hypotension, bleeding, and hepatic encephalopathy, possibly caused by the interventions were recorded according to Common Terminology Criteria for Adverse Events (CTCAE) version 4.0.

### Sample size

We planned to enroll 10 patients per arm, with which we would be able to estimate the percentage of patients who completed the study intervention of 80% to within a 95% confidence interval of 62 and 98% [39, 40]. This study was not powered for a direct comparison between CART and paracentesis.

### Randomization and blinding

Patients were randomly assigned in a 1:1 ratio to either the CART (fast-track) arm or control (waiting list: simple paracentesis) arm. Allocation was done by minimization to balance two potential confounders: hemorrhagic ascites (absent or present) and serum total protein (< 5.5, >  = 5.5 g/dL) [25]. All patients, clinicians, and investigators were aware of the treatment allocation. All clinicians and investigators were blinded to the questionnaire, except for the attending nurse who supported the patients to fill it in.

### Statistical analysis

We summarized the baseline demographics and feasibility outcomes using descriptive statistics. Then, Kaplan–Meier curves were computed for paracentesis-free survival and PRO-paracentesis-free survival following the intention to treat principle. We described adverse events of each arm. As a feasibility trial, these endpoints were summarized descriptively, with no formal statistical comparisons between arms.

Next, we tested the differences in the abdominal distension NRS score, total score of ESAS:AM, and abdominal circumference of patients who completed the observation at 3 time points: day 1, day 2, and the day of the next paracentesis (or the day closest to the next paracentesis day of the days when ESAS:AM was measured), using one-way repeated measures ANOVA. As we explored whether these measures reflected the effect of ascites drainage and recurrence of ascites, we did not perform a statistical comparison between arms.

Then, we visualized the changes in outcome measures for each patient, and classified them into stable increase and random changes. We assessed the agreement between the day of receiving the next paracentesis and the first day of the patient’s request for paracentesis: agreement with the request: received paracentesis within 2 days of the first request, delayed paracentesis: received paracentesis 2 days or more after the first request, early paracentesis: received paracentesis before the request.

All statistical analyses were performed using R, version 3.5.3 (R Core Team 2019, Vienna, Austria). All *P*-values were 2-sided. A *P*-value of < 0.05 was considered significant. Survival analyses were performed using the ‘‘survival’’ package.

## Results

### Study flow

**Fig. 1:**
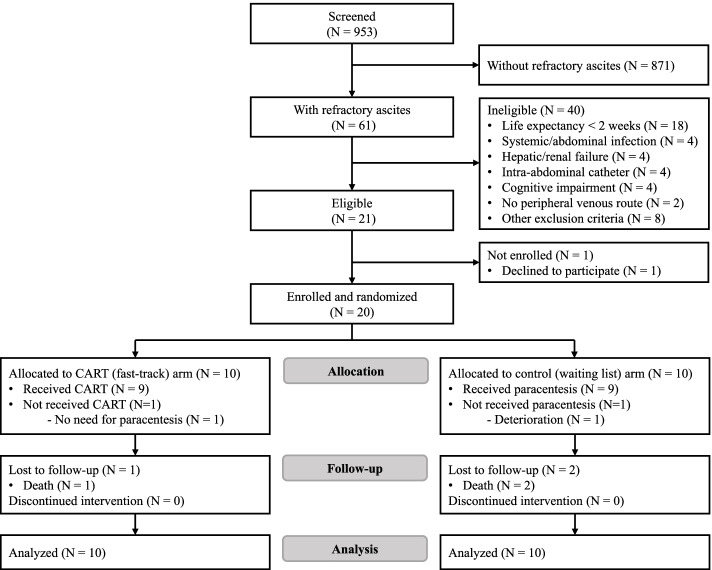
CONSORT flow diagram. Abbreviation: CART Cell-free and concentrated ascites reinfusion theraphy

Figure 1 shows the CONSORT flow diagram for this study. Among the 953 patients screened, 61 (6.4%, 95% CI: 4.9–8.1) patients had refractory malignant ascites and 21 (2.2%, 95% CI: 1.4–3.3) were eligible. The main reasons for ineligibility of 40 excluded patients with refractory malignant ascites included a life expectancy of two weeks or less (*N* = 18), fever or infection (4), hepatic or renal failure (4), intra-abdominal catheter placement (4), and cognitive impairment (4). As one patient declined, a total of 20 (95.2%, 95% CI: 76.2–99.9) of the eligible patients were enrolled and randomized.

### Patients’ characteristics

The patients’ baseline characteristics are shown in Table 1. The median age was 71.5, and 80% were female. All patients had an ECOG performance status of 3 or 4. The most frequent primary tumor site was the pancreas (25%), followed by the gastrointestine (20%), and liver and bile duct (15%). Massive liver metastasis existed in 30% of patients. Opioids were administered to 45% of patients. Ascites was hemorrhagic in 30% of patients. The median previous paracentesis interval was 7 days. The median abdominal distension NRS score and total ESAS:AM score were 5.5 and 35.5, respectively.

**Table 1:** Patients’ characteristics

Variable	CART *N* = 10	Control *N* = 10	Total *N* = 20
***Patients’ characteristics***			
Age (median [range])	71.0 [63.0, 87.0]	73.5 [41.0, 95.0]	71.5 [41.0, 95.0]
Sex, female (%)	8 (80)	8 (80)	16 (80)
Institution (%)			
A	5 (50)	8 (80)	13 (65)
B	3 (30)	1 (10)	4 (20)
C	2 (20)	1 (10)	3 (15)
D	0 (0)	0 (0)	0 (0)
ECOG performance status (%)			
0–2	0 (0)	0 (0)	0 (0)
3	8 (80)	8 (80)	16 (80)
4	2 (20)	2 (20)	4 (20)
Primary tumor site (%)			
Pancreas	4 (40)	1 (10)	5 (25)
Gastrointestinal	1 (10)	3 (30)	4 (20)
Liver, bile duct	2 (20)	1 (10)	3 (15)
Gynecological	0 (0)	2 (20)	2 (10)
Urological	0 (0)	1 (10)	1 (5)
Others	3 (30)	2 (20)	5 (25)
Liver metastasis (%)			
Absent	6 (60)	5 (50)	11 (55)
Not massive	1 (10)	2 (20)	3 (15)
Massive	3 (30)	3 (30)	6 (30)
Lung metastasis (%)			
Absent	10 (100)	9 (90)	15 (75)
Present	0 (0.0)	1 (10)	5 (25)
***Co-treatment***			
Opioid (%)			
0 mg/day	7 (70)	4 (40)	11 (55)
< 60 mg/day	1 (10)	4 (40)	5 (25)
> = 60 mg/day	2 (20)	2 (20)	4 (20)
Diuretics (%)			
Absent	6 (60)	5 (50)	10 (50)
Present	4 (40)	5 (50)	10 (50)
Hydration (mL, median [range])	0 [0, 300]	0 [0, 500]	0 [0, 500]
***Ascites feature***			
Appearance			
Serous	7 (70)	7 (70)	14 (70)
Hemorrhagic	3 (30)	3 (30)	6 (30)
Chylous	0 (0)	0 (0)	0 (0)
Serum-ascites albumin gradient (g/dL, median [range])	1.40 [0.60, 1.80]	1.00 [0.20, 2.20]	1.30 [0.20, 2.20]
Previous time-to-next paracentesis (days, median [range])	6.0 [4.0, 15.0]	5.0 [3.0, 13.0]	7.00 [3.00, 15.00]
***Laboratory findings***			
Total protein (g/dL, median [range])	5.55 [4.90, 7.30]	5.60 [4.60, 7.20]	5.60 [4.60, 7.30]
Albumin (g/dL, median [range])	1.95 [1.60, 3.80]	2.50 [1.60, 3.40]	2.30 [1.60, 3.80]
Total bilirubin (mg/dL, median [range])	0.68 [0.40, 2.50]	0.65 [0.30, 1.50]	0.68 [0.30, 2.50]
Creatinine (mg/dL, median [range])	0.65 [0.33, 2.00]	0.94 [0.67, 1.35]	0.84 [0.33, 2.00]
***Physical symptoms***			
Abdominal distension NRS (median [range])	5.0 [2.0, 10.0]	6.0 [1.0, 9.0]	5.5 [1.0, 10.0]
ESAS:AM total score (median [range])	37.0 [10.0, 67.0]	30.0 [15.0, 73.0]	35.5 [10.0, 73.0]

*Abbreviations*: *CART* Cell-free and concentrated ascites reinfusion therapy, *ECOG* Eastern Cooperative Oncology Group, *NRS* Numerical rating scale, *ESAS:AM* Edmonton Symptom Assessment System: Ascites Modification

### Treatment parameters

Details of allocated treatments are shown in Table 2. The median drained ascites volume was 3,200 mL in the CART arm and 2,500 mL in the control arm. In the CART arm, the median concentration of removed ascites was 10.9 times, and the median reinfused albumin volume was 12.6 g.

**Table 2:** Treatment parameters

Treatment parameter	CART (*N* = 9)	Control (*N* = 9)
Drained ascites volume (median [range])	3200 [1900, 4100]	2500 [1500, 4700]
Concentration (times, median [range])	10.9 [6.1, 34.2]	NA
Reinfused albumin (g, median [range]) ^a^	12.6 [3.2, 32.8]	NA

^a^Multiplied concentration of reinfused ascites albumin by reinfused ascites volume

*Abbreviation*: *NA* Not applicable

### Feasibility

A total of 18 patients received and completed study intervention (90%, 95% CI: 68.3–98.8), while 2 patients did not receive allocated treatments because there was either no need for paracentesis or deterioration. Paracentesis-free survival events occurred in 17 patients (85%, 95% CI: 62.1–96.8): 15 patients underwent the next paracentesis; two patients died during the observational period due to tumor progression; one was censored after the final observation.

All patients in the control (waiting list) arm, except for 3 patients who died or deteriorated, received CART after the study.

### Secondary endpoints

**Fig. 2:**
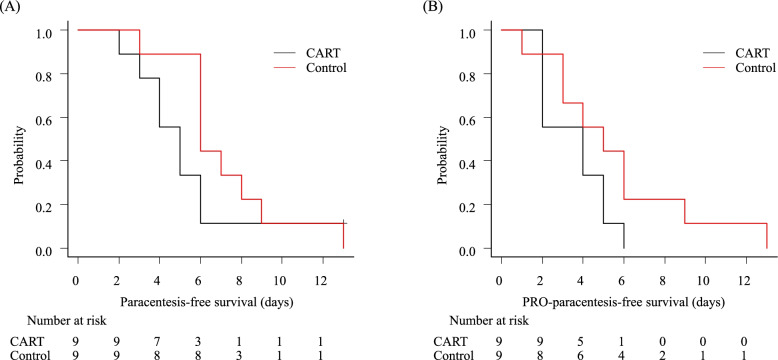
Paracentesis interval. Kaplan-Meier curves for paracentesis- free survival (**A**) and patient's request on the questionnaire for paracentesis (PRO-paracentesis)-free survival (**B**)

Median paracentesis-free survival was 5 days (95% CI: 2–6) in the CART arm and 6 days (95% CI: 3–9) in the control arm (Fig. 2A). Median PRO-paracentesis-free survival was 4 days (95% CI: 2–5) in the CART arm and 5 days (95% CI: 1–9) in the control arm (Fig. 2B).

No severe adverse event was observed in either arm. Grade 1 fever occurred in one patient in the CART arm. No patients experienced hypotension, bleeding, hepatic encephalopathy, or other adverse events.

### Exploratory analyses of outcome measures

#### Changes in physical symptoms and abdominal circumference

In 15 patients who received allocated treatment and completed follow-up observation, the mean abdominal distension NRS score significantly changed over time: 5.5 (95%CI: 3.9 – 7.0) before allocated treatment, 3.2 (1.8 – 4.6) the day after treatment, and 5.5 (4.0 -6.9) before the next paracentesis (*p* = 0.0008, Supplemental Fig. 1A). Similarly, the mean total score of ESAS:AM significantly changed over time: 37.6 (95%CI: 25.7 – 49.5) before allocated treatment, 28.2 (15.6 – 40.8) the day after treatment, and 43.0 (27.4 – 58.6) before the next paracentesis (*p* = 0.033, Supplemental Fig. 1B).

The change in the mean abdominal circumference was not significant: 88.5 cm (95%CI: 82.3 – 94.8) before allocated treatment, 86.7 (80.3 – 93.2) the day after allocated treatment, and 88.2 (82.8 – 93.6) before the next paracentesis (*p* = 0.11, Supplemental Fig. 1C). Median change of abdominal circumference after the treatment was -2.5 cm (range: -11, + 4.0).

## Changes of outcome measures in individual patients

A total of 67% of patients showed a steady increase in the abdominal distension NRS score after paracentesis, as expected (cases 3–6, 9, 11–17), although 11% of patients showed random changes within days (case 8, 10; Supplemental Fig. 2). The pattern of changes in abdominal circumference showed wide variations within patients (Supplemental Fig. 2).

We did not observe any difference in the distribution of agreement between receiving and requesting the next paracentesis between arms (Supplemental Fig. 3). Among the 15 patients who actually received the next paracentesis, a total of 11 patients (73%) received paracentesis within 2 days from their first request for the next paracentesis. While 2 patients received paracentesis more than 2 days after their request, 2 other patients received paracentesis before their request.

## Discussion

This is, to our best knowledge, the first randomized trial of reinfusion of concentrated ascitic fluid, or CART, for patients with malignant ascites. In this study, we revealed that a fast-track, randomized controlled trial (RCT) was feasible for patients with refractory malignant ascites.

The most important finding of this study was that most of the enrolled patients completed study interventions. The high completion rate suggests that this study design is feasible. In addition, high rates of consent and outcome event occurrence further support the feasibility. The high consent rate may be because all participants have a chance to receive active treatment with a fast-track design [34]. Actually, 16 of 20 enrolled patients received CART as the allocated treatment or treatment after the study. On the other hand, only 6.3% of screened patients suffered refractory ascites requiring repeated paracentesis, and thus just 2.2% of all screened patients were eligible in this study. This is consistent with a previous study reporting that intractable malignant ascites accounted for 6% of patients admitted to a hospice [10]. Thus, we believe the fast-track RCT of CART vs. simple paracentesis for patients with refractory malignant ascites is feasible, although patient recruitment might be difficult.

Other important findings were the features of potential outcome measures. While the ideal outcomes of malignant ascites treatments may be long-term symptom control, quality of life, or survival, we believe that surrogate outcomes that can be evaluated in a short period are favorable to increase the feasibility of studies involving patients with a poor condition [13, 14]. Although some clinical trials used the paracentesis interval as an endpoint of malignant ascites treatments, this outcome measure is open to risk of bias related to unblinding, i.e., physicians treating patients allocated to intervention may be reluctant to perform the next paracentesis [13, 14]. We assessed the following measures as alternative or complementary endpoints. First, a patient’s request on the questionnaire for paracentesis (PRO-paracentesis)-free survival is a patient-reported outcome showing good consistency with paracentesis-free survival. It could confirm the value of paracentesis-free survival as a valid and reliable endpoint. It also could be used to verify whether paracentesis-free survival was affected by the physician’s intention. Second, patient-reported physical symptom scales: abdominal distension NRS and the total ESAS:AM score, could be outcome measures. Although some variations existed, they generally improved and steadily increased after paracentesis. We might be able to compare the time-to-baseline score or cumulative scores during certain observation periods in a future study. Finally, abdominal circumference did not change significantly over time. As this result might be due to the small sample size or small drainage volume, further studies are needed to evaluate whether abdominal circumference is useful as an endpoint of ascites treatments.

Of note was that CART may be a safe treatment. In contrast to early studies of reinfusion of concentrated ascitic fluid reporting that about 40% of patients suffered fever, mild fever occurred in one patient (10%) in the CART arm in this study [23, 24]. This was consistent with recent studies reporting that fever occurred in 5—12% of patients treated with CART for malignant ascites [25–28]. A potential interpretation is that technical improvement of the procedure and the membranes used for filtration and concentration might contribute to the safety of this treatment [26, 41]. However, the efficacy and safety of CART should be confirmed in a future randomized trial.

Strengths of this study included: sample size calculation for the feasibility endpoint, [39, 40] adoption of fast-track design which was recommended in a palliative care setting [33, 34], and exploration of potential patient-reported outcome measures of ascites treatments. This study had several limitations. First, the sample size was small. We believe, however, we could safely say that fast-track RCT of CART and paracentesis were feasible because the sample size of 20 was calculated for the primary feasibility endpoint, i.e., the percentage of patients who completed the study intervention. As a feasibility trial, secondary endpoints were summarized descriptively, with no formal statistical comparisons between the arms. Second, we did not strictly set the drainage volumes in each arm, although the primary responsible palliative care physician decided them according to the national clinical guideline in which 1 to 3 L of paracentesis is proposed [3]. As a larger volume of drainage may be associated with a longer paracentesis interval, we may need to standardize or specify the drainage volume in both arms to compare the efficacy of treatments in a future study. Third, further studies are needed to evaluate the efficacy and safety of CART in combination with chemotherapy, while this study included patients who received no further anticancer treatments to eliminate their effect on the outcomes [29, 30]. Fourth, as we measured ESAS:AM every other day, findings of ESAS:AM might lack reliability. Finally, the method to measure the abdominal circumference was not strictly standardized. More detailed standardization of the procedure may be necessary in a future study.

## Conclusion

A fast-track RCT of CART and paracentesis for patients with malignant ascites is feasible. The efficacy and safety of CART should be assessed in future trials. Patient’s request on the questionnaire for paracentesis-free survival may be a complementary outcome measure with paracentesis-free survival in future trials.

## Supplementary Information

**Additional file 1.**

## Data Availability

The datasets generated and analyzed during the current study are not publicly available due to limitations of ethical approval involving the patient data and anonymity but are available from the corresponding author on reasonable request.

## Abbreviations

CART: Cell-free and concentrated ascites reinfusion therapy
PRO-paracentesis-free survival: Patient’s request on the questionnaire for paracentesis-free survival
ESAS:AM: Edmonton Symptom Assessment System: Ascites Modification
NRS: Numerical rating scale
CTCAE: Common Terminology Criteria for Adverse Events
