# Supplementary material for: Feasibility of a fast-track randomized controlled trial of cell-free and concentrated ascites reinfusion therapy for patients with refractory malignant ascites
Source: BMC Cancer. 2022 Feb 28;22:218. doi: 10.1186/s12885-022-09336-3 (PMC8883725; doi:10.1186/s12885-022-09336-3)
Supplement: Supplementary file 1 — Additional file 1. [file 12885_2022_9336_MOESM1_ESM.pptx]

## Slide 1
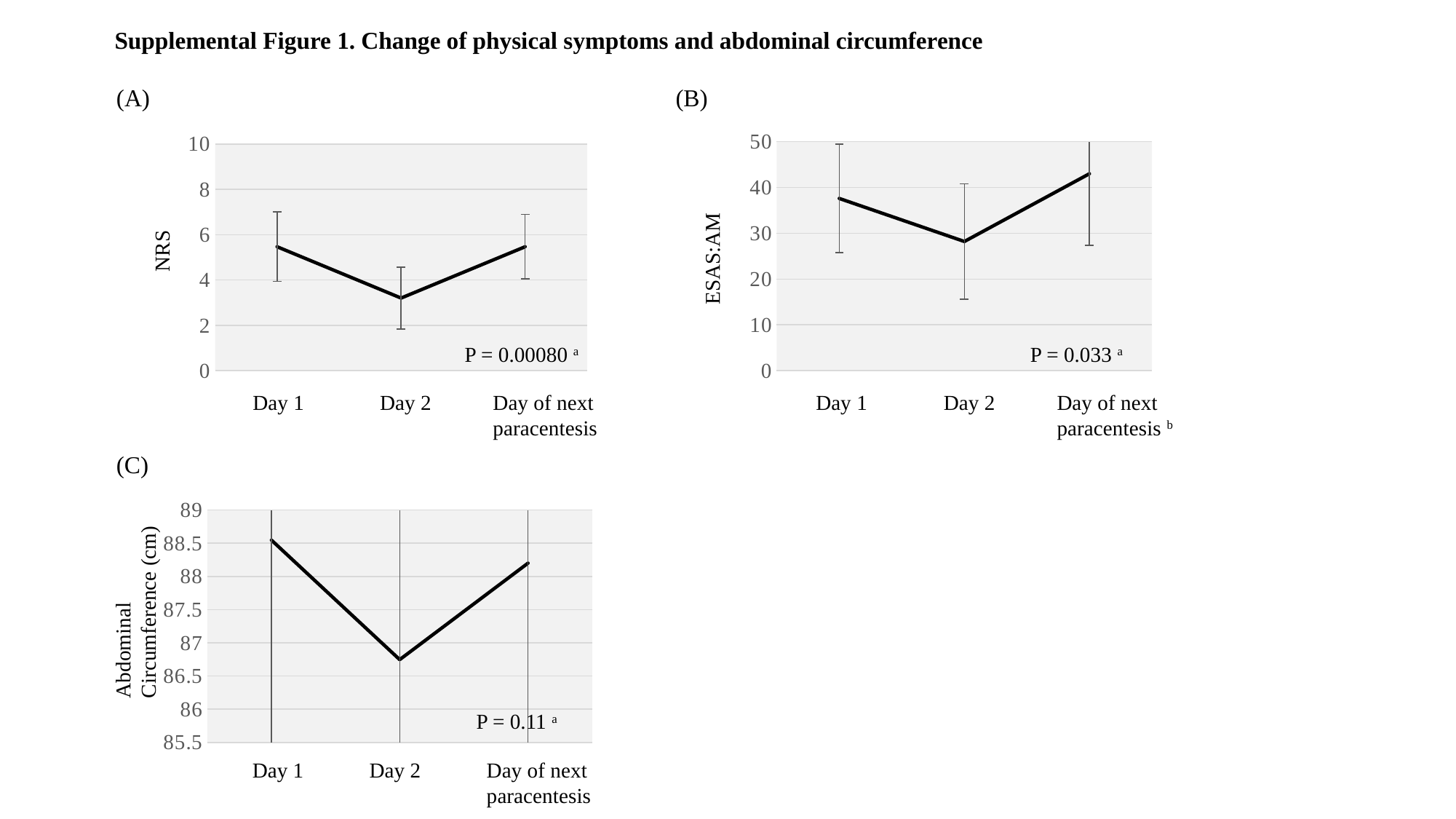

Supplemental Figure 1. Change of physical symptoms and abdominal circumference
### Chart
| Category | |
|---|---|(B)
ESAS:AM
Day 1
Day of next paracentesis b
Day 2
(A)
### Chart
| Category | |
|---|---|Day 1
Day of next paracentesis
Day 2
NRS
P = 0.00080 a
P = 0.033 a
(C)
### Chart
| Category | |
|---|---|Day 1
Day 2
Day of next paracentesis
Abdominal Circumference (cm)
P = 0.11 a

## Slide 2
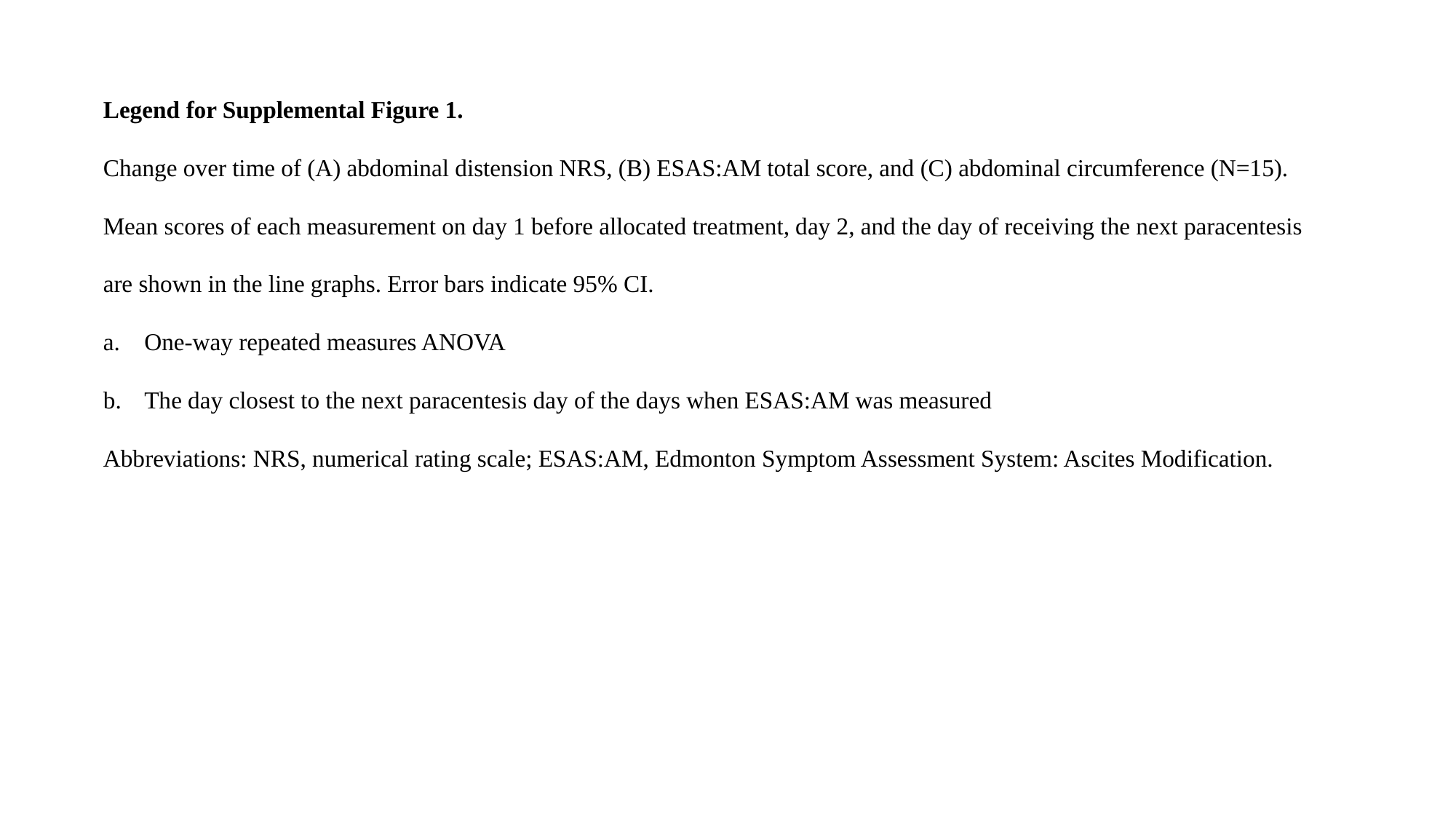

Legend for Supplemental Figure 1.
Change over time of (A) abdominal distension NRS, (B) ESAS:AM total score, and (C) abdominal circumference (N=15). Mean scores of each measurement on day 1 before allocated treatment, day 2, and the day of receiving the next paracentesis are shown in the line graphs. Error bars indicate 95% CI.
One-way repeated measures ANOVA
The day closest to the next paracentesis day of the days when ESAS:AM was measured
Abbreviations: NRS, numerical rating scale; ESAS:AM, Edmonton Symptom Assessment System: Ascites Modification.

## Slide 3
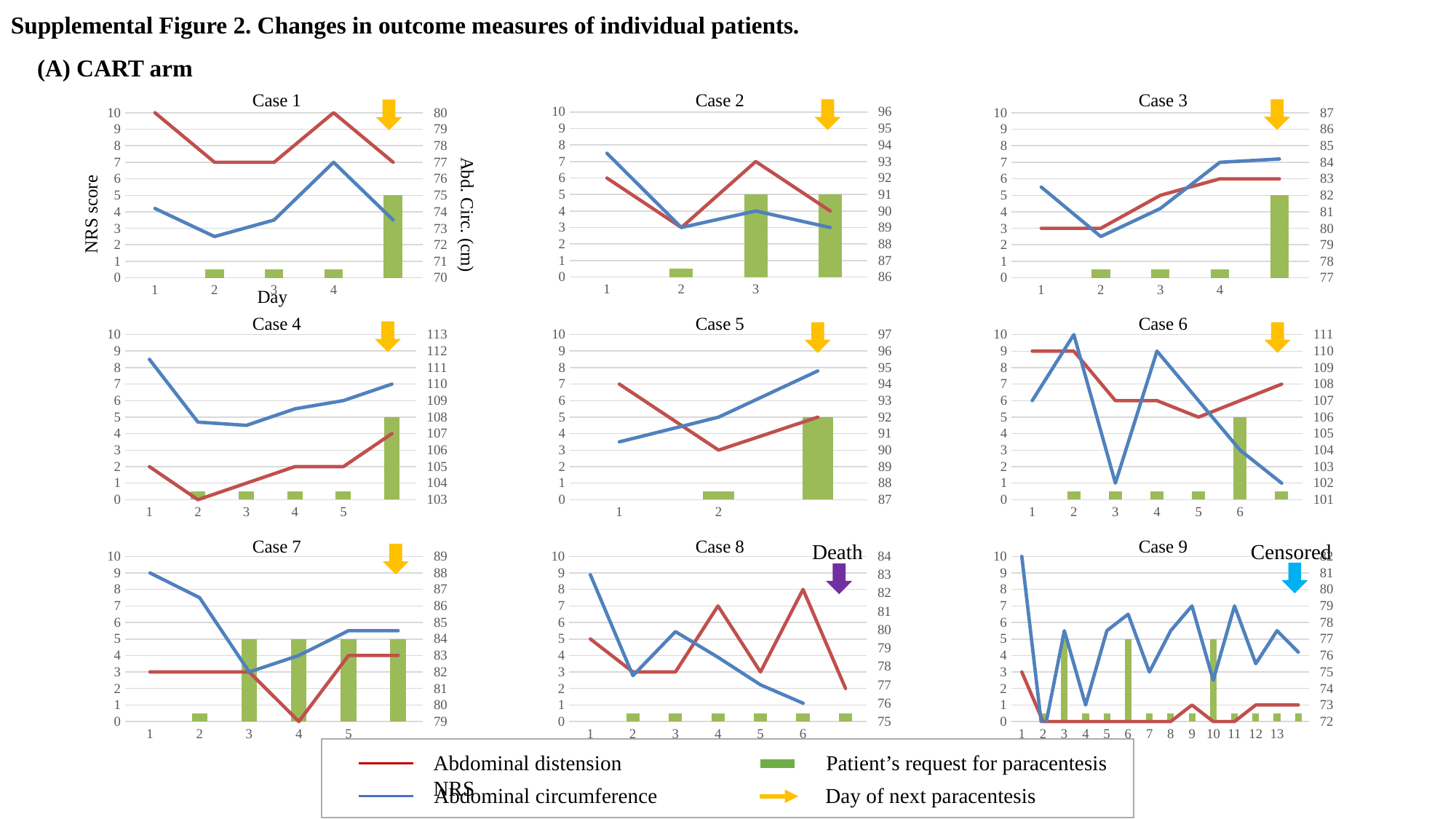

Supplemental Figure 2. Changes in outcome measures of individual patients.
(A) CART arm
Case 1
Case 2
Case 3
### Chart
| Category | | | |
|---|---|---|---|
### Chart
| Category | | | |
|---|---|---|---|
### Chart
| Category | | | |
|---|---|---|---|NRS score
Abd. Circ. (cm)
Day
Case 4
Case 5
Case 6
### Chart
| Category | | | |
|---|---|---|---|
### Chart
| Category | | | |
|---|---|---|---|
### Chart
| Category | | | |
|---|---|---|---|
Case 7
Case 8
Case 9
Censored
Death
### Chart
| Category | | | |
|---|---|---|---|
### Chart
| Category | | | |
|---|---|---|---|
### Chart
| Category | | | |
|---|---|---|---|
Abdominal distension NRS
Patient’s request for paracentesis
Abdominal circumference
Day of next paracentesis

## Slide 4
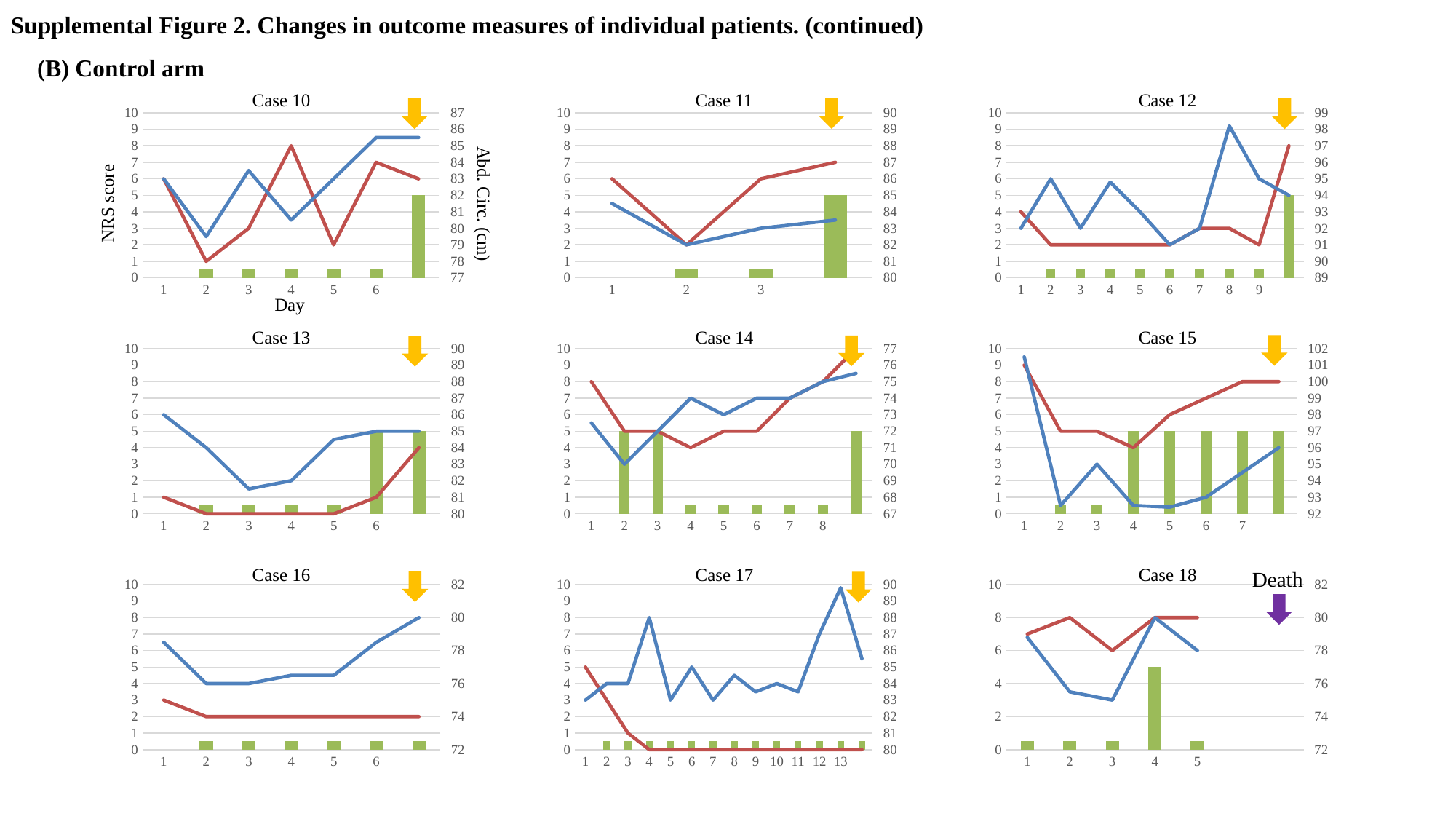

Supplemental Figure 2. Changes in outcome measures of individual patients. (continued)
(B) Control arm
Case 10
Case 11
Case 12
### Chart
| Category | | | |
|---|---|---|---|
### Chart
| Category | | | |
|---|---|---|---|
### Chart
| Category | | | |
|---|---|---|---|NRS score
Abd. Circ. (cm)
Day
Case 13
Case 14
Case 15
### Chart
| Category | | | |
|---|---|---|---|
### Chart
| Category | | | |
|---|---|---|---|
### Chart
| Category | | | |
|---|---|---|---|
Case 16
Case 17
Case 18
Death
### Chart
| Category | | | |
|---|---|---|---|
### Chart
| Category | | | |
|---|---|---|---|
### Chart
| Category | | | |
|---|---|---|---|

## Slide 5
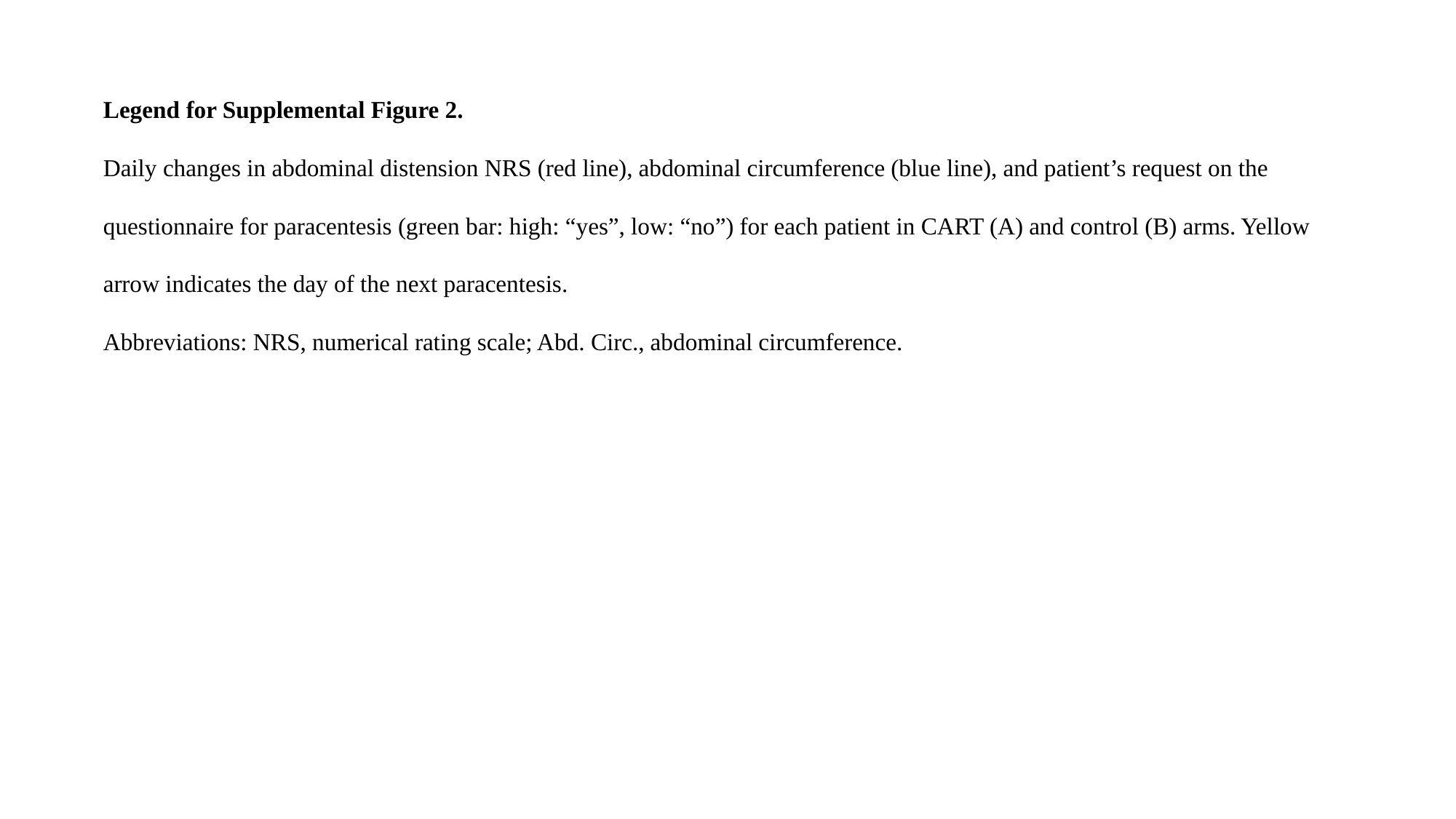

Legend for Supplemental Figure 2.
Daily changes in abdominal distension NRS (red line), abdominal circumference (blue line), and patient’s request on the questionnaire for paracentesis (green bar: high: “yes”, low: “no”) for each patient in CART (A) and control (B) arms. Yellow arrow indicates the day of the next paracentesis.
Abbreviations: NRS, numerical rating scale; Abd. Circ., abdominal circumference.

## Slide 6
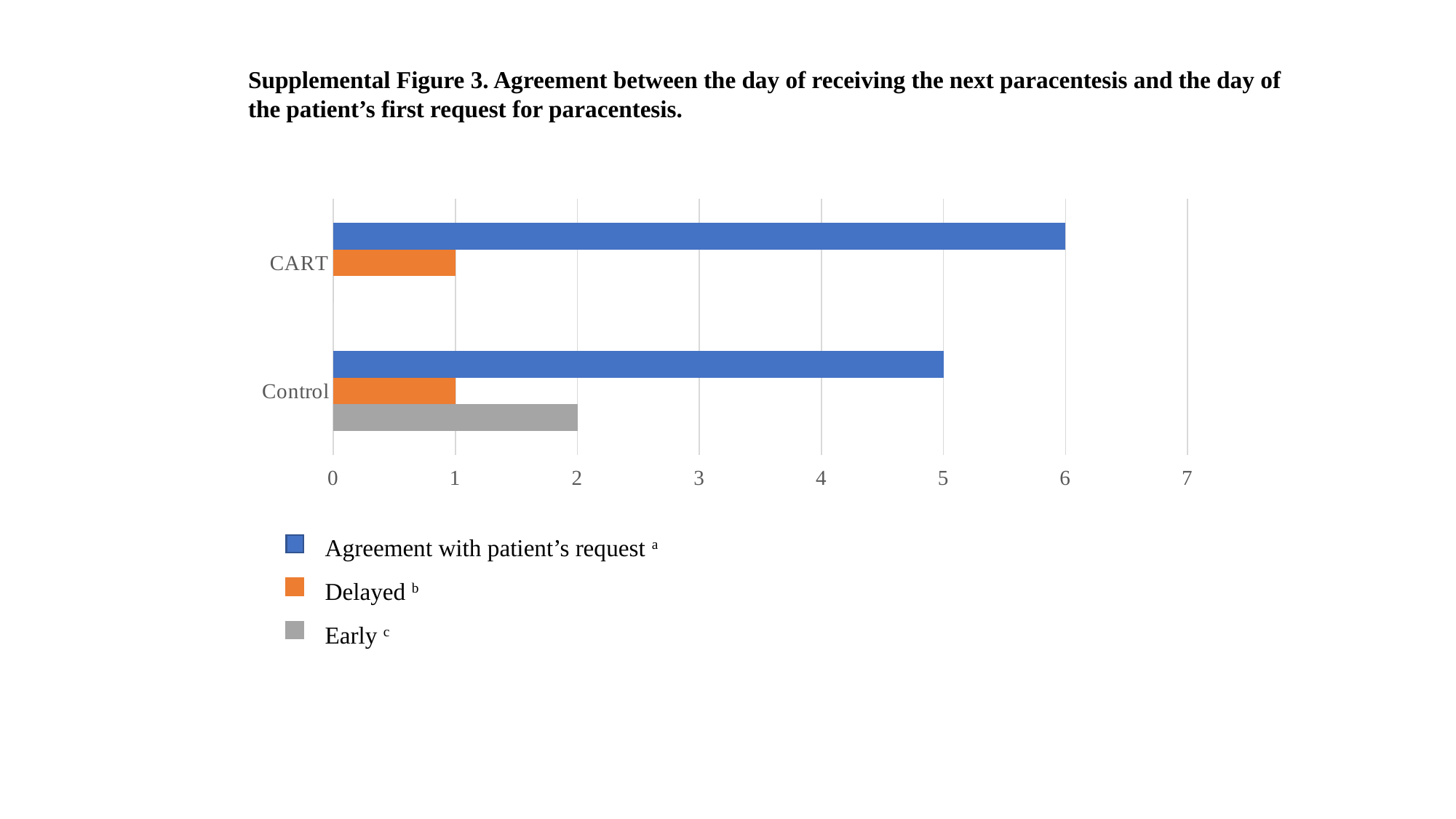

Supplemental Figure 3. Agreement between the day of receiving the next paracentesis and the day of the patient’s first request for paracentesis.
### Chart
| Category | Consistent | Delayed | Early |
|---|---|---|---|
| CART | 6.0 | 1.0 | 0.0 |
| Control | 5.0 | 1.0 | 2.0 |Agreement with patient’s request a
Delayed b
Early c

## Slide 7
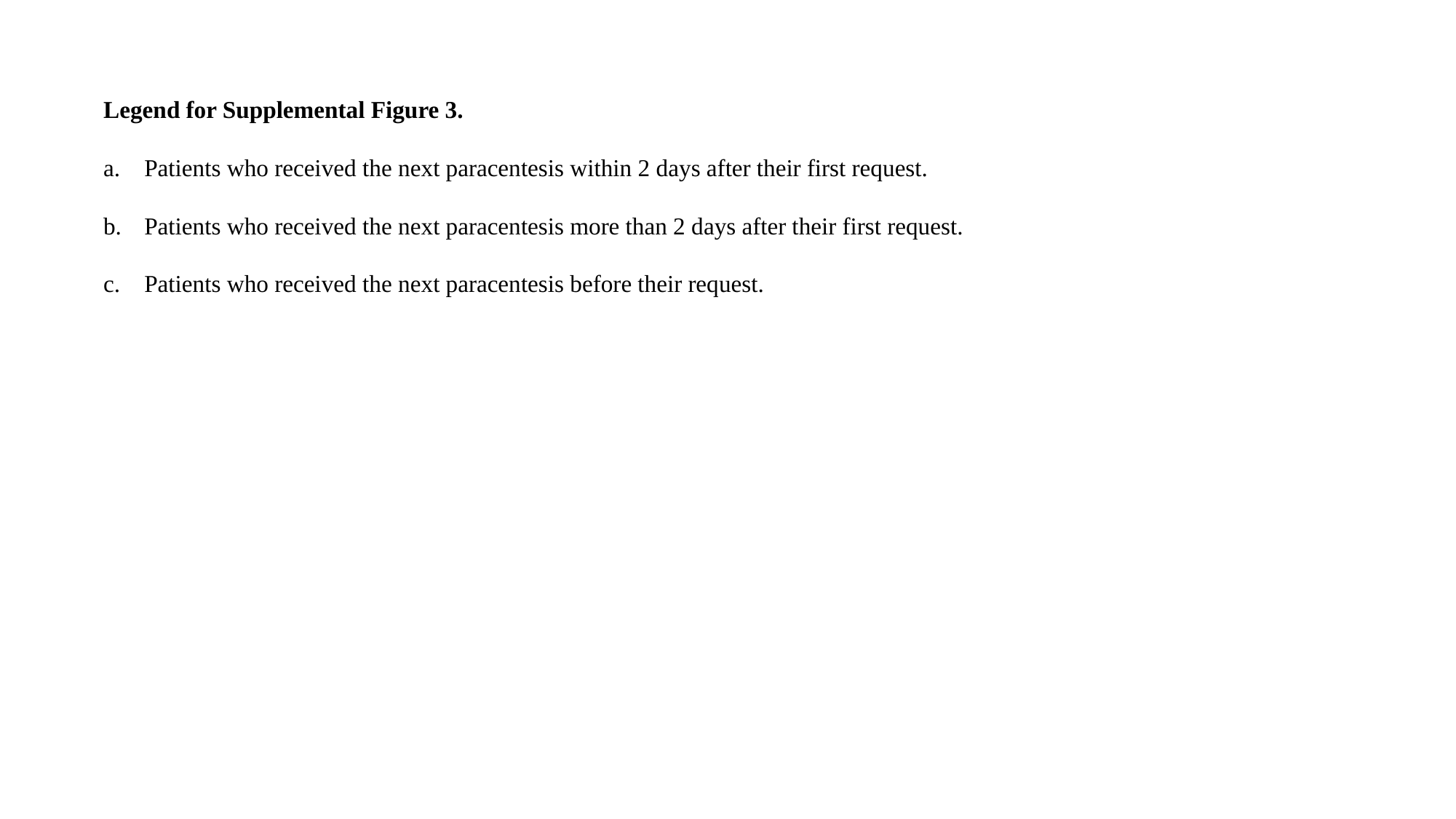

Legend for Supplemental Figure 3.
Patients who received the next paracentesis within 2 days after their first request.
Patients who received the next paracentesis more than 2 days after their first request.
Patients who received the next paracentesis before their request.
